# Dynamic Changes in Serum AFP, Albumin and ALBI after Radioembolization in Patients with Unresectable Hepatocellular Carcinoma with High or Low Alpha-Fetoprotein Levels

**DOI:** 10.47829/jjgh.2025.11105

**Published:** 2026-08-13

**Authors:** BI Carr, HG Gozukara Bag, R Kutlu, V Ince, S Yilmaz

**Affiliations:** 1 Liver Transplant Institute, Inonu University Faculty of Medicine, 44280, Malatya, Turkey; 2 Department of Biostatistics, Inonu University Faculty of Medicine, 44280, Malatya, Turkey; 3 Department of Radiology and Liver Transplant Institute, Inonu University Faculty of Medicine, 44280, Malatya, Turkey

**Keywords:** Hepatocellular Carcinoma, Radioembolization, Biomarker, Prognosis

## Abstract

**Background:**

Yttrium-90 trans arterial radioembolization (TARE) is an established locoregional therapy for unresectable hepatocellular carcinoma (HCC). Baseline biomarkers are widely used for prognosis, but the clinical significance of post-TARE changes in tumour and liver-function biomarkers remains uncertain. ALBI and related measures have emerged as objective prognostic indices of hepatic reserve and HCC.

**Aim:**

To identify early post-TARE biomarker changes associated with overall survival.

**Methods:**

We retrospectively analysed 276 nonsurgical patients with HCC who underwent TARE and had available survival data. Changes in routinely measured tumour, inflammatory, hematologic, and liver-function variables were assessed at 1, 3, 6, and 12 months, when available.

**Results:**

Biomarker changes were evident by 1 month, particularly in AFP, albumin, and ALBI grade, in patients with both low and high baseline AFP. Changes in PALBI, mALBI, GGT, CRP, and bilirubin were also observed. Increased or stable albumin was associated with longer survival at all assessed time points in both baseline AFP strata. Decreased AFP was associated with longer survival consistently in patients with high baseline AFP and at later time points in patients with low baseline AFP. Early bilirubin decreases were also associated with longer survival. Patients whose NPS phenotype decreased had substantially longer median survival than those with increased phenotype.

**Conclusions:**

Early post-TARE changes in albumin and AFP were associated with overall survival in this retrospective cohort. These findings support future prospective validation of dynamic biomarker changes.

## 2. Introduction

Locoregional therapy has long been central to the nonsurgical management of hepatocellular carcinoma (HCC), initially through trans arterial chemoembolization and, more recently, through yttrium-90 trans arterial radioembolization (TARE) [1–3]. TARE is increasingly used because of its favourable safety profile and feasibility in selected patients with portal vein tumour thrombosis [4].

The role of TARE in HCC management continues to evolve. It may be used as frontline locoregional therapy, for downstaging before liver transplantation, or in combination with kinase inhibitors or immune-checkpoint inhibitors [5,6]. In this retrospective study, we evaluated tumour- and liver-related biomarker responses after TARE according to baseline alpha-fetoprotein (AFP) level, because routinely available response markers are limited in patients with low AFP. We also examined associations between biomarker changes and overall survival. Changes in AFP, albumin, C-reactive protein (CRP), and ALBI were detectable as early as 1 month after TARE.

## 3. Methods

We retrospectively studied 276 adults with nonmetastatic HCC that was not amenable to curative surgery. All patients underwent TARE and were followed for changes in tumour markers, inflammatory markers, complete blood counts, liver-function tests, and survival.

Demographic and clinical characteristics, baseline and follow-up laboratory values, and abdominal computed tomography (CT) findings were analysed. Variables included AFP, liver-function tests, complete blood counts, maximum tumour diameter, tumour number, and the presence or absence of macroscopic portal vein tumour thrombosis. ALBI grade was calculated using the standard published formula. NPS phenotype was calculated using the NPS web tool (https://apkatos.github.io/webpage_nps).

HCC was diagnosed histologically or noninvasively according to European Association for the Study of the Liver criteria, based on characteristic imaging findings in a hepatic lesion >2 cm in the setting of cirrhosis. TARE was performed during an inpatient admission, and patients were generally observed for 24 hours after the procedure.

Eligibility criteria were age >18 years; HCC considered unsuitable for definitive surgical treatment; no extrahepatic metastases; lung shunt fraction <20%; Eastern Cooperative Oncology Group performance status <3; adequate hematologic, renal, and hepatic function for intervention; and informed consent for radioembolization.

### 3.1. Radioembolization Procedures

Treatment followed published guidance [7] and our previously reported protocol [8]. Pretreatment angiographic mapping of the abdominal aorta and hepatic arteries was performed. After selective arterial injection of technetium-99m macroaggregated albumin, planar anterior and posterior lung and liver imaging was followed by SPECT (before 2006) or SPECT/CT. Radioembolization used Yttrium-90 resin microspheres (SIR-Spheres; Sirtex Medical, North Sydney, Australia) or glass microspheres (Thera Sphere; Boston Scientific, Marlborough, MA, USA). The treating interventional radiologist used individualized dosimetry based on estimated absorbed doses to tumour and nontumorous liver.

### 3.2. Statistical Analysis

Categorical variables are reported as number (percentage) and were compared using Pearson chi-square tests, with exact tests when appropriate. When an overall test was significant, Bonferroni-adjusted pairwise comparisons identified the categories responsible for the difference; superscript letters denote these comparisons. Overall survival was estimated using the Kaplan-Meier method and compared with log-rank tests. Univariate Cox proportional-hazards models were used to estimate hazard ratios (HRs) and 95% confidence intervals. All tests were two-sided, and P<0.05 was considered statistically significant. Analyses were performed using IBM SPSS Statistics for Windows, version 26.0 (IBM Corp., Armonk, NY, USA).

## 4. Results

### 4.1. Serum Biomarker Changes Post-TARE According to Baseline AFP

Patients were stratified by baseline AFP level (<100 vs >=100 IU/mL), as previously reported [8]. Biomarker changes were assessed at 1, 3, 6, and 12 months after TARE when measurements were available (Table 1A). In the high-AFP subgroup, AFP responses were considered to be >50% decrease from baseline and were found in 56.6%, 60.0%, 68.8%, and 77.8% of patients at 1, 3, 6, and 12 months, respectively. Corresponding proportions in the low-AFP subgroup were 27.3%, 42.4%, 43.0%, and 48.7%. AFP increases were also observed at each time point.

Because albumin and ALBI-based indices are established prognostic measures in HCC [9–14], we evaluated changes in albumin (Table 1B), ALBI grade (Table 1C), PALBI grade (Table 1D), and mALBI grade (Table 1E) within each AFP stratum. Albumin responses were considered to be an increase of >10% from baseline and were observed in 17.3%−32.1% of patients with low baseline AFP and 7.6%−15.4% of patients with high baseline AFP across the four follow-up time points.

ALBI grade improved (decreased) in 14.9%−26.2% of patients with low baseline AFP and 8.3%−12.8% of patients with high baseline AFP (Table 1C). PALBI and mALBI grades also improved in both AFP strata (Tables 1D and 1E).

Changes in blood GGT (Table 1F), CRP (Table 1G), total bilirubin (Table 1H), and platelet count (Table 1I) were also examined. GGT has been reported as an HCC biomarker [15,16], especially in patients with low AFP [17]. GGT responses were considered to be a decrease of >50% from baseline and were found in 13.1%−29.8% of patients with low baseline AFP and 13.6%−33.3% of patients with high baseline AFP. CRP, an inflammatory and prognostic biomarker in HCC [18–20], decreased in some patients, although the number of available CRP measurements was small. Total bilirubin decreased in 16.1%−29.8% of patients with low baseline AFP and 12.8%−17.9% of patients with high baseline AFP. Blood platelet increases are a reflection of portal hypertension improvement in patients with cirrhosis [21,22] and occurred in a minority of patients and were more frequent in the high-AFP group at 12 months.

Parallel analyses of the platelet-to-lymphocyte ratio, neutrophil-to-lymphocyte ratio, neutrophil count, and lymphocyte count did not identify significant changes in either baseline AFP stratum (data not shown).

### 4.2. AFP Changes and Survival Post-TARE

Table 2 presents survival according to AFP change within baseline AFP strata. Among patients with baseline AFP >=100 IU/mL, a >50% AFP decrease was associated with longer survival than stable or increasing AFP at each assessed time point. In the low-AFP subgroup, survival did not differ significantly at the 1- or 3-month assessments but was longer among AFP responders at 6 and 12 months. In a subgroup with baseline AFP of 1,000–5,000 IU/mL, responders also had longer survival at several time points. Because these analyses classify patients by biomarker values measured post-TARE treatment, the results should likely be interpreted as landmark prognostic associations rather than treatment effects.

### 4.3. Albumin, ALBI, GGT, Bilirubin, Platelets and CRP in Relation to Survival Post-TARE

Biomarkers that changed post-TARE (Table 1) were examined in relation to survival post-TARE treatment (Table 3), separately within the low- and high baseline AFP strata. At each assessment, increased albumin was associated with longer survival than decreased albumin in both AFP strata. The magnitude of the unadjusted median-survival differences was often twofold or greater, and the corresponding univariate HRs were statistically significant.

Improvement in ALBI grade was generally associated with numerically longer survival than worsening ALBI grade, but most comparisons were not statistically significant. Significant differences were limited to selected time points and subgroups (Table 3B).

GGT changes were also not consistently associated with survival (Table 3C). Lower total bilirubin was associated with longer survival at early assessments in both AFP strata (Table 3D). Platelet changes were not associated with significant HRs (Table 3E). CRP measurements were available for relatively few patients, but lower CRP was associated with longer survival, reaching statistical significance only at the 6-month assessment (Table 3F).

### 4.4. Associations between AFP Changes and Changes in Other Biomarkers

Table 4 shows whether changes in albumin, ALBI, mALBI, PALBI, GGT, and CRP differed among AFP response categories at 6 months. In the low baseline AFP subgroup, AFP response was associated with albumin increase (41.3%; P=0.001), ALBI-grade improvement (39.1%; P=0.003), and mALBI-grade improvement (45.7%; P=0.001). PALBI, GGT, and CRP changes were not significantly associated with AFP response. In the high baseline AFP subgroup, AFP response was associated with albumin increase (21.2%; P=0.044), whereas associations with the other biomarkers were not statistically significant.

### 4.5. Changes in Network Phenotyping Strategy Post-TARE

Prognostic parameters for HCC have traditionally been used as single factors, such as AFP, PVT or tumour size, for the tumour contribution to prognosis, and as combinations, such as Child-Pugh score or recently, ALBI for the liver contributions. These were then superseded by a limited number of factors together, in recognition of both the liver and tumour contributions. These included ITA.LI.CA, BCLC and JIS amongst many other combinations [23, 24]. We recently integrated 17 common demographic, liver and tumour parameters into a network that yielded 25 discrete survival-associated HCC phenotypes, called the Network phenotyping Strategy or NPS [25,26]. Phenotype 1 patients had the best biology and prognosis, and conversely, phenotype 25 patients had the worst biology. We arbitrarily chose a >3 phenotype decreases to indicate a response to therapy and >3 phenotype increase to indicate disease progression. Anything in between was labelled as stable disease. We took the same approach as in Table 2, but using the combination of 17 integrated clinical parameters (NPS) to examine the effects of TARE on changes in phenotype (Table 5). Using these TARE-response criteria, Table 5A shows that 20.8% of patients had an NPS phenotype decrease or response to TARE, 66.6% were stable and 13% had progression. Examples of responder and progressor phenotype changes are shown in Table 5B. The 3 response categories had differences in survival. The responders (decrease in phenotype) had a median survival of 37.07 months, stable patients had a median survival of 21.37 months and progressors (increase in phenotype) had a median survival of 5.47 months (Table 5C). Specific examples of patient survival in relation to phenotype change post-TARE are shown in Table 5B. It shows 2 patients with phenotype 10 who responded to TARE and survived for 37.07 and 39.33 months respectively (NPS Responder column, left). However, a patient with the same phenotype 10 who progressed (worse phenotype) survived of 5.47 months (NPS Progressor column, right). Similarly for baseline NPS Phenotype 12, there were 7 patients with baseline phenotype 12 who had a response and 4 patients who also had a baseline phenotype of 12 who progressed. The baseline phenotype 12 responder patients had a mean survival of 60.34 months and the progressor patients from the same baseline phenotype 12 had a mean survival of 5.08 months (Table 5C, bottom 2 lines). This illustrates the limitations of relying solely on baseline characteristics rather than response to therapy, similar to the results in Table 2 for AFP response, or Table 3 for albumin response, since the integrated biological changes using NPS after TARE showed a more complex picture.

## 5. Discussion

We previously showed, like others before us, that baseline ALBI grade, serum albumin and AFP levels relate to survival. In this study we extend that, by showing that both ALBI grade, albumin and AFP levels all change with TARE treatment, and we examined the consequences of those changes for survival.

The main findings in this study are that liver and tumour serum marker responses to therapy may be more important prognosticators for patients treated with TARE than their baseline levels. ALBI and especially serum albumin changes are candidate prognosis markers for HCC patients with low baseline AFP levels. The results focus attention on the role of albumin in HCC biology and furthermore suggest that patients with low or high baseline AFP levels may possibly have variant diseases, rather than simply differences in a parameter level.

We previously found in a small cohort of TARE-treated HCC patients who were followed for 3 months, that there was a high percent of both AFP and GGT responders after TARE in the total cohort, and unexpected improvement in liver function tests in about 20–40% of patients, depending on the liver parameter. We also found both a similar decrease in ALBI grade and an increase in serum albumin levels in response to TARE (8). In the current study, we extended our previous observations to a larger cohort with measurements over a longer time period and measured the survival consequences of changes in liver function tests and HCC markers in greater detail. We did this in part by following and comparing marker changes in patients with either high or low baseline AFP levels.

A major problem in HCC management is that 40–60% of patients are AFP negative, depending on the series. Therefore, new HCC markers of disease response or progression are needed, especially for that large proportion of low AFP patients. Although other markers such as AFP-L3 (lens culinaris agglutinin-reactive fraction of AFP) and des-gamma-carboxyprothrombin or DCP (also known as PIVKA-2 for protein induced by vitamin K absence in older literature) have been studied, it is unclear how useful they are for management decisions.

We found here that patients with high baseline serum AFP levels (>100 IU/mL) also had a good response rate (56.6% of patients at 1 month to 77.8 % of patients at 12 months post-TARE). Patients with low baseline serum AFP levels (<100 IU/mL) had similar, but lower responses (27.3% of patients at 1 month to 48.7% of patients at 12 months), as seen in Table 1A. Interestingly and usefully, this response (decrease in AFP level) was clearly measurable by 1-month post-TARE, meaning that it must have started even earlier. The associated survivals (Table 2) were significantly different between the responders and progressors at every time point that was measured in the high baseline AFP subgroup. This finding was similar for the low baseline serum AFP patients, but only at 6 and 12 months after TARE, not earlier. Perhaps this lag in response relates to differences in biology with HCC patients with lower AFP levels also have having slower growth and responses. Thus, median survival in HCC progressors for low versus high baseline AFP patients was 20.7 versus 4.27 months, 22.57 versus 8.37 months, and 16.83 versus 13.20 months for data at 1-, 3- and 6-months post-TARE, respectively. We further tested the relationships of AFP responses to survival in that subset with very high baseline AFP levels >1000 IU/mL (Table 2, bottom group) and found their survival (16.6, 21.43 and 25.47 months at 3, 6 and 12 months post-TARE) to be similar to the responder patients with merely high (>100 IU/mL) or even to low AFP level patients (same Table 2). Thus, a major finding in this study is that AFP response post-TARE seems to be key for prognosis, regardless of baseline AFP levels.

In contrast to the AFP responses (decrease), the albumin responses (percentage of patients with 10% or more increase in serum level) were more striking in patients with low than in patients with high baseline AFP levels. Thus, low baseline AFP patients had a range of increased albumin from 17.3% to 32.1% of patients at 1–12 months after TARE, and for high AFP patients, albumin increased in range from 7.6% to 15.4% of patients (Table 1B). The responses for ALBI, PALBI and mALBI (decrease in grade) were similar to albumin responses, and were similarly greater for the low baseline AFP compared with the high baseline AFP patients for every time point (Tables 1C, 1D and 1E). Responses in GGT (decrease in serum levels) were also similar for patients with high or low serum AFP values (i.e non significant p-values), shown in Table 1F. Decreases in the inflammatory marker CRP as well as in total bilirubin levels were also found in a greater percentage of low versus the high baseline AFP patients, but not significantly (Tables 1G and 1H). A major problem with the CRP data was insufficient patient follow-up CRP records. Platelets increased similarly for both low and high AFP patients at 1, 3 and 6 months, shown in Table 1I.

Thus, responses to TARE resulted in improvements, as manifested in increases for albumin, and decreases for GGT and total bilirubin and all of ALBI, PALBI and mALBI grades. The striking aspect of these results was the greater percent of patients with low rather than high AFP levels for each parameter. Patients with low and high AFP were compared in adjacent columns in Table 1, since high AFP is the predominant universally confirmed HCC-specific prognosticator so far, and thus results for other parameters needed to be compared in low and high AFP patients. The interest of these results is that they suggest albumin and ALBI to be HCC patient post-TARE response parameters in low AFP patients (and possibly also serum GGT and CRP levels).

Survival results for patients who had increased or stable serum albumin levels post-TARE were striking, as they were significantly longer than for patients with a decrease in albumin levels at every time point and regardless of whether they had a low or high baseline serum AFP level, and had significant hazard ratios for survival at each time point (Table 3A). The trend for improvement in survival with decrease (improvement) in ALBI compared to an increase in ALBI was similar to the results for albumin, but several of the comparisons in survival were not significant, despite large absolute differences in months of survival between TARE response subgroups (Table 3B). Thus, changes in albumin rather than changes in ALBI seemed to be more useful for survival in this study. There were very similar, but non-significant findings for PALBI and mALBI (not shown). Decreases in serum GGT levels also did not translate into significant survival differences. However, improvement (decrease) in total bilirubin levels was, unsurprisingly, associated with significant increase in survival compared to patients who had an increase in total bilirubin, regardless of baseline levels of AFP. No hazard ratios were significant for platelet changes and survival.

In Tables 1–3, parameter changes and survivals were related to baseline high or low AFP levels and survival was shown to significantly relate to changes in levels of AFP, albumin and ALBI grade. In Table 4A, the relationships of AFP changes post-TARE to changes in other parameters were examined in patients with low baseline AFP levels. Patients in whom AFP decreased (responded) post-TARE also had significant responses (increase) in albumin levels in 41.3% of patients, and they also had responses (decrease) in ALBI grade in 39.1% of patients and decrease in mALBI grade in 45.7% of patients. This may be particularly useful for clinical management in patients with low baseline AFP levels. Similar significant changes occurred only in albumin levels in patients with high baseline AFP levels (Table 4B). Phenotype decreases (response) or increases (progression) were also found in the 17 parameter NPS phenotype model (Table 5) and, as for AFP, what mattered for survival was the changes in phenotype in response to TARE.

The association between albumin and survival may reflect hepatic reserve, systemic inflammation, tumour burden, treatment response, or a combination of these factors. Experimental studies have also suggested direct effects of albumin on HCC proliferation and invasion [32–35], and successful treatment of chronic viral hepatitis may increase albumin levels [36–38]. Nevertheless, the present study cannot determine whether albumin is causal, a surrogate of disease severity, or both. The observed post-TARE increases in albumin and decreases in bilirubin may reflect changes in tumour burden, hepatic inflammation, or functional liver volume [7,8,42].

The principal finding is that early changes in albumin and AFP post-TARE were associated with survival in both baseline AFP strata. Dynamic measures may provide information beyond baseline values, particularly in patients with low baseline AFP, for whom routinely available tumour-response biomarkers are limited. However, these observational associations do not establish that improving albumin or AFP itself improves survival. The results require prospective validation using time-dependent multivariable analyses. The NPS findings similarly suggest that integrated phenotypic change may have prognostic value, but they should currently be regarded as exploratory. The rapid changes in AFP level and in albumin and ALBI score in many patients, as early as 1 month post TARE, suggest that there must be rapid changes in the tumour biology, as reflected in the significantly longer survival in responding patients, none of whom had additional surgical therapies. This has practical significance for liver transplant centres, since surgical treatment is typically conditional on levels of AFP (and perhaps in future on other markers). Increasingly, subsets of patients with poor prognosis markers such as portal vein thrombosis or large tumour size, have been reported to have improved survival when levels of some biomarkers such as GGT are low [27,28]. Response to therapy is a dynamic phenotypic change and since its assessment is empirical, follow up measurements are needed for more accurate prognostic consideration. The NPS results which integrate 17 parameters, show that phenotype can change post-TARE and not just the AFP, and be significant for survival. Others have also reported ALBI changes early after immune therapy initiation [29,30].

A major question that arises from these observations on the prognostic usefulness of both baseline [31] and changes in albumin and ALBI post-TARE, is why does a liver function parameter predict HCC survival and what are the mechanisms underlying the improvement? Is it just that HCC patients often die a liver death, likely related to parenchymal destruction by the growing tumour mass(es)? Or additionally, might inflammatory liver disease promote HCC growth and invasion and if so, how? Albumin might function passively as a known reflection of inflammation. There are also several reports on the direct experimental effects of albumin on HCC growth or invasion [32–35]. Furthermore, successful treatment of the etiological hepatitis has been shown to result in increased albumin levels [36–38], which might in turn influence the biology or development of the associated HCC [39–41]. A radiation effect or a consequence of tumour damage from the TARE may be involved in these liver function improvements, which have been noted elsewhere [7,8,42].

Strengths of this study include the relatively large single-centre TARE cohort and the evaluation of several routinely available biomarkers over multiple time points. The usefulness of this is that patients are often from resource-challenged countries and can live at a distance from a tertiary centre, so that follow-up scans to measure objective tumour changes are frequently not done. Important limitations include the retrospective design; missing and progressively fewer biomarker measurements over time; use of univariate rather than multivariable Cox models and no external validation. Therefore, the findings should be considered hypothesis-generating. Albumin supplementation or other attempts to manipulate albumin cannot be recommended on the basis of these data. Prospective validation will be needed before dynamic biomarkers can be used for clinical guidelines, although some, such as major AFP responses to oncology therapies are already in use in downstaging to liver transplantation.

## Figures and Tables

**Table 1:** Changes in AFP and other biomarkers over time post-TARE.

		Baseline AFP		
A.AFP changes		IU/mL 100>	IU/mL 100≤	p
.mo 1	Decreased	a(27.3) 42	b(56.6) 69	0.001>
	Stable	(30.5) 47	(21.3) 26	
	Increased	a(42.2) 65	b(22.1) 27	
.mo 3	Decreased	(42.4) 53	(60) 45	0.055
	Stable	(19.2) 24	(13.3) 10	
	Increased	(38.4) 48	(26.7) 20	
.mo 6	Decreased	a(43) 46	b(68.8) 33	0.012
	Stable	(25.2) 27	(12.5) 6	
	Increased	(31.8) 34	(18.8) 9	
.mo 12	Decreased	a(48.7) 37	b(77.8) 28	0.007
	Stable	a(21.1) 16	b(2.8) 1	
	Increased	(30.3) 23	(19.4) 7	
		Baseline AFP		
B. Albumin changes		<100 IU/mL	≥100 IU/mL	p
1 mo.	Decreased	62 (36.9)a	59 (44.7)b	0.039
	Stable	77 (45.8)	63 (47.7)	
	Increased	29 (17.3)	10 (7.6)	
3 mo.	Decreased	45 (31.9)a	41 (48.8)b	0.005
	Stable	63 (44.7)	36 (42.9)	
	Increased	33 (23.4)a	7 (8.3)b	
6 mo.	Decreased	27 (22.5)	19 (36.5)	0.567
	Stable	53 (44.2)	25 (48.1)	
	Increased	40 (33.3)	8 (15.4)	
12 mo.	Decreased	26 (31.0)	14 (35.9)	0.173
	Stable	31 (36.9)	19 (48.7)	
	Increased	27 (32.1)	6 (15.4)	
		Baseline AFP		
C. ALBI grade changes		<100 IU/mL	≥100 IU/mL	p
1 mo.	Decreased	25 (14.9)	11 (8.3)	0.017
	Stable	106 (63.1)	74 (56.1)	
	Increased	37 (22.0)a	47 (35.6)b	
3 mo.	Decreased	24 (17.0)	10 (11.9)	0.001
	Stable	94 (66.7)a	41 (48.8)b	
	Increased	23 (16.3)a	33 (39.3)b	
6 mo.	Decreased	25 (20.8)	6 (11.8)	0.371
	Stable	63 (52.5)	30 (58.8)	
	Increased	32 (26.7)	15 (29.4)	
12 mo.	Decreased	22 (26.2)	5 (12.8)	0.222
	Stable	43 (51.2)	22 (56.4)	
	Increased	19 (22.6)	12 (30.8)	
		Baseline AFP		
D. PALBI grade changes		<100 IU/mL	≥100 IU/mL	p
1 mo.	Decreased	27 (16.2)	15 (11.4)	0.500
	Stable	95 (56.9)	80 (61.1)	
	Increased	45 (26.9)	36 (27.5)	
3 mo.	Decreased	31 (22.3)	12 (14.3)	0.164
	Stable	71 (51.1)	41 (48.8)	
	Increased	37 (26.6)	31 (36.9)	
6 mo.	Decreased	33 (27.7)	10 (20.0)	0.118
	Stable	55 (46.2)	19 (38.0)	
	Increased	31 (26.1)	21 (42.0)	
12 mo.	Decreased	26 (31.3)	5 (12.8)	0.091
	Stable	39 (47.0)	23 (59.0)	
	Increased	18 (21.7)	11 (28.2)	
		Baseline AFP		
E. mALBI grade changes		IU/mL 100 >	IU/mL 100≤	p
.mo 1	Decreased	(17.2) 29	(9.8) 13	0.006
	Stable	(53.0) 89	(43.2) 57	
	Increased	a(29.8) 50	b(47.0) 62	
.mo 3	Decreased	(22.0) 31	(13.1) 11	0.001
	Stable	a(51.8) 73	b(36.9) 31	
	Increased	a(26.2) 37	b(50.0) 42	
.mo 6	Decreased	(25.0) 30	(13.7) 7	0.260
	Stable	(40.0) 48	(45.1) 23	
	Increased	(35.0) 42	(41.2) 21	
.mo 12	Decreased	(29.8) 25	(15.4) 6	0.173
	Stable	(36.9) 31	(51.3) 20	
	Increased	(33.3) 28	(33.3) 13	
		Baseline AFP		
F. GGT changes		<100 IU/mL	≥100 IU/mL	p
1 mo.	Decreased	22 (13.1)	18 (13.6)	0.553
	Stable	76 (45.2)	67 (50.8)	
	Increased	70 (41.7)	47 (35.6)	
3 mo.	Decreased	36 (25.7)	22 (25.9)	0.895
	Stable	60 (42.9)	34 (40)	
	Increased	44 (31.4)	29 (34.1)	
6 mo.	Decreased	31 (25.8)	17 (33.3)	0.149
	Stable	52 (43.3)	14 (27.5)	
	Increased	37 (30.8)	20 (39.2)	
12 mo.	Decreased	25 (29.8)	13 (33.3)	0.417
	Stable	36 (42.9)	12 (30.8)	
	Increased	23 (27.4)	14 (35.9)	
		Baseline AFP		
G. CRP changes		<100 IU/mL	≥100 IU/mL	p
1 mo.	Decreased	10 (29.4)	5 (22.7)	0.858
	Stable	10 (29.4)	7 (31.8)	
	Increased	14 (41.2)	10 (45.5)	
3 mo.	Decreased	13 (46.4)	5 (31.3)	0.107
	Stable	8 (28.6)	2 (12.5)	
	Increased	7 (25)	9 (56.3)	
6 mo.	Decreased	8 (25)	2 (20)	1.000
	Stable	10 (31.3)	3 (30)	
	Increased	14 (43.8)	5 (50)	
12 mo.	Decreased	6 (28.6)	1 (20)	1.000
	Stable	9 (42.9)	2 (40)	
	Increased	6 (28.6)	2 (40)	
		Baseline AFP		
H. Total bilirubin changes		<100 IU/mL	≥100 IU/mL	p
1 mo.	Decreased	27 (16.1)	17 (12.8)	0.099
	Stable	82 (48.8)	53 (39.8)	
	Increased	59 (35.1)	63 (47.4)	
3 mo.	Decreased	30 (21.3)	9 (10.7)	0.122
	Stable	58 (41.1)	41 (48.8)	
	Increased	53 (37.6)	34 (40.5)	
6 mo.	Decreased	29 (24.1)	10 (19.6)	0.653
	Stable	41 (34.2)	16 (31.4)	
	Increased	50 (41.7)	25 (49.0)	
12 mo.	Decreased	25 (29.8)	7 (17.9)	0.381
	Stable	24 (28.6)	13 (33.3)	
	Increased	35 (41.7)	19 (48.7)	
		Baseline AFP		
I. Platelet # changes		<100 IU/mL	≥100 IU/mL	p
1 mo.	Decreased	29 (17.4)	17 (12.9)	0.430
	Stable	112 (67.1)	89 (67.4)	
	Increased	26 (15.6)	26 (19.7)	
3 mo.	Decreased	22 (15.8)	8 (9.5)	0.398
	Stable	88 (63.3)	56 (66.7)	
	Increased	29 (20.9)	20 (23.8)	
6 mo.	Decreased	24 (20.2)	6 (12)	0.245
	Stable	71 (59.7)	29 (58)	
	Increased	24 (20.2)	15 (30)	
12 mo.	Decreased	20 (24.1)	5 (12.8)	0.004
	Stable	51 (61.4)	18 (46.2)	
	Increased	12 (14.5)a	16 (41)b	

Different superscript letters indicate statistically significant differences between column proportions.

For each biomarker, values are the number (percentage) of patients with a decrease, stable value, or increase. AFP is reported in IU/mL. Abbreviations: AFP, alpha-fetoprotein; ALBI, albumin-bilirubin grade; CRP, C-reactive protein; GGT, gamma-glutamyl transferase; mALBI, modified ALBI grade; PALBI, platelet-albumin-bilirubin grade.

**Table 2:** AFP changes and overall survival post-TARE.

Change	Patients	AFP	Overall surviv-(al (months Mean±SE	Overall surviv-(al (months Median±SE	Log-rank p-value	(.HR (95% C.I	HR p-value
.From baseline to 1 mo	All	(Decreased (n=95	26.67±3.52	16.07±3.00	0.325	reference	
		(Stable (n=65	18.73±2.80	9.20±2.28		(0.920–1.827) 1.296	0.138
		(Increased (n=85	22.86±3.06	13.20±3.98		(0.788–1.500) 1.087	0.609
	Baseline AFP<100	(Decreased (n=31	27.87±4.07	20.33±3.62	0.694	reference	
		(Stable (n=40	24.93±4.01	17.10±3.35		(0.733–2.135) 1.251	0.411
		(Increased (n=58	29.44±4.06	20.70±2.56		(0.661–1.799) 1.090	0.735
	Baseline AFP≥100	(Decreased (n=64	22.51±3.82	11.37±3.27	0.001>	reference	
		(Stable (n=25	8.42±1.70	5.53±2.33		(1.318–3.522) 2.155	0.002
		(Increased (n=27	8.64±2.08	4.27±0.43		(1.383–3.567) 2.221	0.001
.From baseline to 3 mo	All	(Decreased (n=73	35.36±4.23	21.10±2.21	0.321	reference	
		(Stable (n=31	28.40±6.18	17.10±4.99		(0.829–2.155) 1.337	0.233
		(Increased (n=65	26.93±3.92	18.70±3.76		(0.881–1.904) 1.295	0.189
	Baseline AFP<100	(Decreased (n=33	33.72±5.18	20.33±3.18	0.930	reference	
		(Stable (n=21	29.07±4.99	20.87±3.61		(0.584–2.098) 1.107	0.755
		(Increased (n=46	32.94±4.88	22.57±3.10		(0.646–1.862) 1.097	0.732
	Baseline AFP≥100	(Decreased (n=40	31.42±5.41	21.10±2.50	0.001>	reference	
		(Stable (n=10	15.73±8.98	5.47±0.79		(1.345–6.291) 2.909	0.007
		(Increased (n=19	9.90±1.80	8.37±2.85		(1.588–5.559) 2.971	0.001
.From baseline to 6 mo	All	(Decreased (n=57	43.19±4.92	27.07±2.29	0.010	reference	
		(Stable (n=30	29.88±4.20	22.70±5.59		(0.828–2.364) 1.399	0.209
		(Increased (n=43	21.42±2.26	16.83±3.82		(1.281–3.297) 2.055	0.003
	Baseline AFP<100	(Decreased (n=28	44.76±5.28	32.03±5.97	0.003	reference	
		(Stable (n=24	35.23±4.64	28.90±4.44		(0.725–2.868) 1.443	0.296
		(Increased (n=34	22.12±2.54	16.83±4.93		(1.465–5.184) 2.756	0.002
	Baseline AFP≥100	(Decreased (n=29	35.49±6.41	25.47±3.18	0.001>	reference	
		(Stable (n=6	8.48±0.94	8.53±1.16		(3.729–32.889) 11.074	0.001>
		(Increased (n=4	16.27±2.62	13.20±2.73		(0.872–5.329) 2.155	0.096
.From baseline to 12 mo	All	(Decreased (n=49	47.11±5.54	30.43±2.59	0.003	reference	
		(Stable (n=13	43.56±6.66	41.03±6.60		(0.332–1.764) 0.765	0.530
		(Increased (n=30	26.78±3.44	23.03±3.26		(1.307–3.863) 2.247	0.003
	Baseline AFP<100	(Decreased (n=24	42.43±5.84	29.07±6.84	0.018	reference	
		(Stable (n=12	46.18±6.68	53.93±7.79		(0.247–1.686) 0.646	0.372
		(Increased (n=23	28.67±4.59	25.40±3.81		(1.018–4.093) 2.041	0.045
	Baseline AFP≥100	(Decreased (n=25	46.16±7.73	30.43±3.07	0.026	reference	
		(Stable (n=1					
		(Increased (n=7	21.85±2.84	23.03±9.30		(1.085–7.072) 2.770	0.033

Change	AFP≥1000 IU/mL	Overall survival (months) Mean±SE	Overall survival (months) Median±SE	Log-rank p-value	HR (95% C.I.)	HR p-value
From baseline to 1 mo.	Decreased (n=37)	14.57±2.54	8.87±2.11	0.016	reference	
	Stable (n=22)	7.79±1.88	4.00±1.15		1.829 (1.054–3.175)	0.032
	Increased (n=19)	7.17±2.27	3.47±0.53		2.075 (1.172–3.673)	0.012
From baseline to 3 mo.	Decreased (n=25)	20.27±3.99	16.60±4.771	<0.001	reference	
	Stable (n=7)	6.11±0.78	5.47±0.26		5.746 (2.087–15.825)	0.001
	Increased (n=10)	7.94±1.50	5.53±3.11		3.309 (1.396–7.842)	0.007
From baseline to 6 mo.	Decreased (n=17)	20.68±2.22	21.43±3.84	<0.001	reference	
	Stable (n=6)	8.48±0.94	8.53±1.16		8.115 (2.381–27.657)	0.001
	Increased (n=3)	9.59±1.27	10.27±2.56		6.286 (1.420–27.827)	0.015
From baseline to 12 mo.	Decreased (n=13)	32.20±6.61	25.47±4.28	0.440	reference	
	Increased (n=4)	20.93±4.47	15.93±5.42		1.619 (0.472–5.551)	0.444

AFP, alpha-fetoprotein (IU/mL); CI, confidence interval; HR, hazard ratio.

**Table 3:** Biomarker changes post-TARE in relation to overall survival.

A.Change	Patients	Albumin	Overall survival (months) Mean±SE	Overall survival (months) Median±SE	Log-rank p-value	HR (95% C.I.)	HR p-value
From baseline to 1 mo.	All	Increased+Stable (n=259)	24.30±1.96	13.20±1.70	<0.001	reference	
		Decreased (n=11)	3.78±0.68	3.07±0.57		5.898 (3.123–11.137)	<0.001
	Baseline AFP<100	Increased+Stable (n=140)	28.94±2.72	19.20±1.46	<0.001	reference	
		Decreased (n=2)	3.13±0.40	2.73±NA		13.196 (2.935–59.334)	0.001
	Baseline AFP≥100	Increased+Stable (n=117)	17.24±2.38	8.50±0.92	<0.001	reference	
		Decreased (n=9)	3.93±0.83	3.07±0.85		3.838 (1.880–7.835)	0.001
From baseline to 3 mo.	All	Increased+Stable (n=180)	31.75±2.69	19.83±1.48	<0.001	reference	
		Decreased (n=16)	7.17±1.47	5.27±0.93		4.951 (2.887–8.490)	<0.001
	Baseline AFP<100	Increased+Stable (n=110)	34.37±3.35	20.87±1.73	0.030	reference	
		Decreased (n=5)	12.73±3.63	9.87±2.45		2.653 (1.061–6.634)	0.037
	Baseline AFP≥100	Increased+Stable (n=69)	25.94±4.03	15.43±3.29	<0.001	reference	
		Decreased (n=11)	4.65±0.53	4.00±0.44		9.764 (4.361–21.858)	<0.001
From baseline to 6 mo.	All	Increased+Stable (n=131)	35.07±2.99	23.43±1.66	<0.001	reference	
		Decreased (n=15)	13.11±2.67	9.20±0.62		3.419 (1.932–6.053)	<0.001
	Baseline AFP<100	Increased+Stable (n=90)	34.64±2.79	25.37±2.36	<0.001	reference	
		Decreased (n=7)	13.51±2.68	11.37±1.96		4.295 (1.916–9.625)	<0.001
	Baseline AFP≥100	Increased+Stable (n=40)	27.97±4.90	21.07±3.29	0.026	reference	
		Decreased (n=8)	12.77±4.51	8.40±0.66		2.537 (1.086–5.925)	0.031
From baseline to 12 mo.	All	Increased+Stable (n=89)	43.75±3.77	30.23±1.92	<0.001	reference	
		Decreased (n=13)	17.69±1.99	16.07±1.90		5.271 (2.736–10.155)	<0.001
	Baseline AFP<100	Increased+Stable (n=58)	45.47±4.71	31.17±3.84	<0.001	reference	
		Decreased (n=7)	17.77±2.59	16.07±2.79		5.594 (2.390–13.091)	<0.001
	Baseline AFP≥100	Increased+Stable (n=30)	39.82±6.13	27.83±1.50	<0.001	reference	
		Decreased (n=6)	15.99±1.81	12.2±3.29		5.739 (1.903–17.301)	0 .002

B.Change	Patients	ALBI	Overall survival (months) Mean±SE	Overall survival (months) Median±SE	Log-rank p-value	HR (95% C.I.)	HR p-value
From baseline to 1 mo.	All	Decreased (n=20)	25.78±3.67	24.00±3.00	0.001	reference	
		Stable (n=171)	26.67±2.56	15.33±1.91		1.238 (0.747–2.050)	0.407
		Increased (n=79)	13.83±1.79	6.43±1.81		2.019 (1.188–3.433)	0.009
	Baseline AFP<100	Decreased (n=12)	27.29±5.40	24.00±2.45	0.300	reference	
		Stable (n=97)	30.48±3.31	19.43±1.66		1.092 (0.564–2.113)	0.794
		Increased (n=33)	17.47±2.45	13.67±5.17		1.516 (0.732–3.141)	0.263
	Baseline AFP≥100	Decreased (n=8)	23.34±4.37	19.90±7.68	0.009	reference	
		Stable (n=72)	19.74±3.59	8.53±0.74		1.590 (0.725–3.483)	0.247
		Increased (n=46)	10.16±1.99	3.80±0.34		2.607 (1.170–5.808)	0.019
From baseline to 3 mo.	All	Decreased (n=18)	29.55±4.72	25.63±0.46	0.003	reference	
		Stable (n=124)	34.97±3.43	19.83±1.39		1.093 (0.633–1.888)	0.750
		Increased (n=54)	16.95±2.32	9.47±1.87		1.947 (1.087–3.489)	0.025
	Baseline AFP<100	Decreased (n=10)	36.01±6.88	25.63±10.65	0.197	reference	
		Stable (n=83)	36.40±4.02	20.87±1.83		1.248 (0.596–2.615)	0.557
		Increased (n=22)	20.99±3.87	12.37±5.47		1.888 (0.823–4.330)	0.134
	Baseline AFP≥100	Decreased (n=8)	19.96±3.22	13.20±8.28	0.066	reference	
		Stable (n=40)	30.89±5.86	15.93±5.43		1.064 (0.467–2.427)	0.883
		Increased (n=32)	13.59±2.66	6.80±2.05		1.872 (0.815–4.301)	0.140
From baseline to 6 mo.	All	Decreased (n=14)	34.51±5.56	30.77±4.32	0.071	reference	
		Stable (n=86)	35.01±3.59	23.43±1.90		1.361 (0.677–2.738)	0.387
		Increased (n=45)	24.55±3.63	12.97±2.62		2.024 (0.971–4.222)	0.060
	Baseline AFP<100	Decreased (n=10)	35.22±6.54	25.63±9.27	0.182	reference	
		Stable (n=57)	35.20±3.41	25.37±1.99		1.183 (0.530–2.643)	0.682
		Increased (n=30)	26.23±4.39	16.07±4.27		1.819 (0.777–4.262)	0.168
	Baseline AFP≥100	Decreased (n=4)	26.38±5.38	30.77±0.00	0.265	reference	
		Stable (n=28)	28.25±5.76	19.90±3.47		2.002 (0.469–8.536)	0.348
		Increased (n=15)	16.86±3.64	10.27±0.90		2.991 (0.665–13.449)	0.153
From baseline to 12 mo.	All	Decreased (n=13)	39.85±5.50	38.87±6.81	0.038	reference	
		Stable (n=59)	44.89±4.64	27.93±2.18		1.098 (0.530–2.272)	0.802
		Increased (n=30)	26.95±3.25	18.17±2.53		2.070 (0.949–4.513)	0.067
	Baseline AFP<100	Decreased (n=9)	36.47±4.35	38.87±10.75	0.038	reference	
		Stable (n=38)	48.47±5.75	30.77±5.12		0.845 (0.362–1.973)	0.697
		Increased (n=18)	23.49±2.89	17.17±1.41		2.034 (0.803–5.154)	0.134
	Baseline AFP≥100	Decreased (n=4)	45.63±13.76	30.77±6.47	0.392	reference	
		Stable (n=20)	38.58±7.44	25.87±4.14		1.872 (0.419–8.368)	0.412
		Increased (n=12)	27.95±5.45	19.90±6.35		2.694 (0.578–12.561)	0.207

C.Change	Patients	GGT	Overall survival ((months Mean±SE	Overall survival ((months Median±SE	Log-rank p-value	(.HR (95% C.I	HR p-value
.From baseline to 1 mo	AH	(Decreased (n=34	18.89±3.81	7.80±1.31	0.792	reference	
		(Stable (n=132	23.61±2.65	12.90±2.24		(0.578–1.324) 0.875	0.527
		(Increased (n=104	23.12±2.89	12.37±3.37		(0.566–1.329) 0.868	0.514
	Baseline AFP<100	(Decreased (n=16	22.79±6.31	7.80±0.83	0.838	reference	
		(Stable (n=69	26.19±2.86	18.70±1.56		(0.451–1.568) 0.841	0.586
		(Increased (n=57	29.59±4.32	20.70±2.42		(0.438–1.568) 0.828	0.563
	Baseline AFP≥100	(Decreased (n=17	12.57±3.45	5.53±2.38	0.835	reference	
		(Stable (n=62	18.20±3.62	8.50±1.38		(0.482–1.473) 0.843	0.549
		(Increased (n=47	15.00±2.76	7.93±2.22		(0.491–1.558) 0.875	0.650
.From baseline to 3 mo	AH	(Decreased (n=47	30.30±5.16	15.43±4.48	0.625	reference	
		(Stable (n=88	32.25±3.86	18.17±1.86		(0.584–1.313) 0.876	0.521
		(Increased (n=60	24.22±3.52	19.20±2.21		(0.677–1.597) 1.039	0.860
	Baseline AFP<100	(Decreased (n=27	32.06±5.64	20.70±4.04	0.403	reference	
		(Stable (n=54	33.15±3.78	20.87±2.28		(0.534–1.607) 0.926	0.784
		(Increased (n=33	27.27±4.59	23.30±2.57		(0.719–2.292) 1.283	0.399
	Baseline AFP≥100	(Decreased (n=20	21.56±6.84	7.77±4.62	0.660	reference	
		(Stable (n=33	21.91±4.94	11.60±0.82		(0.439–1.474) 0.804	0.481
		(Increased (n=27	20.74±3.80	13.20±6.17		(0.395–1.425) 0.751	0.380
.From baseline to 6 mo	AH	(Decreased (n=37	27.29±3.45	19.83±2.07	0.002	reference	
		(Stable (n=59	45.78±4.92	25.37±3.97		(0.387–1.041) 0.635	0.072
		(Increased (n=50	20.88±1.88	19.20±3.93		(0.863–2.227) 1.386	0.177
	Baseline AFP<100	(Decreased (n=23	29.48±4.47	19.83±2.02	0.071	reference	
		(Stable (n=45	40.70±4.28	26.73±4.78		(0.367–1.228) 0.671	0.196
		(Increased (n=29	24.51±2.60	20.87±5.22		(0.671–2.287) 1.238	0.494
	Baseline AFP≥100	(Decreased (n=14	21.31±3.79	16.60±7.97	0.067	reference	
		(Stable (n=14	44.02±11.64	21.07±5.91		(0.254–1.581) 0.633	0.327
		(Increased (n=20	15.34±2.02	10.60±0.82		(0.756–3.530) 1.634	0.212
.From baseline to 12 mo	AH	(Decreased (n=25	40.43±6.42	30.77±7.52	0.298	reference	
		(Stable (n=44	41.37±4.33	31.17±4.74		(0.460–1.582) 0.853	0.614
		(Increased (n=33	35.83±4.93	26.97±1.26		(0.709–2.413) 1.308	0.390
	Baseline AFP<100	(Decreased (n=15	28.99±4.22	19.83±8.12	0.193	reference	
		(Stable (n=32	44.34±5.08	31.17±8.42		(0.256–1.074) 0.525	0.078
		(Increased (n=18	44.99±7.42	29.07±1.41		(0.289–1.358) 0.627	0.236
	Baseline AFP≥100	(Decreased (n=10	61.42±12.25	NA	0.048	reference	
		(Stable (n=12	30.17±5.79	25.47±4.10		(0.698–8.361) 2.416	0.164
		(Increased (n=14	22.53±1.99	22.43±1.22		(1.238–12.589) 3.948	0.020
D.Change	Patients	T.Bil	Overall survival ((months Mean±SE	Overall survival ((months Median±SE	Log-rank p-value	(.HR (95% C.I	HR p-value
.From baseline to 1 mo	AH	(Decreased (n=27	28.21±6.11	19.43±6.98	0.001>	reference	
		(Stable (n=132	26.86±2.22	18.70±1.71		(0.588–1.476) 0.931	0.762
		(Increased (n=112	14.88±2.19	5.97±0.91		(1.236–3.117) 1.962	0.004
	Baseline AFP<100	(Decreased (n=11	19.61±4.19	19.83±5.71	0.001>	reference	
		(Stable (n=78	32.88±3.08	23.30±2.40		(0.277–1.020) 0.532	0.057
		(Increased (n=53	20.20±3.70	10.00±3.02		(0.579–2.163) 1.119	0.738
	Baseline AFP≥100	(Decreased (n=15	31.09±9.73	10.97±1.70	0.001>	reference	
		(Stable (n=53	17.79±2.60	11.60±1.56		(0.761–2.855) 1.474	0.250
		(Increased (n=59	9.77±2.19	4.30±0.81		(1.525–5.693) 2.946	0.001
.From baseline to 3 mo	AH	(Decreased (n=24	30.89±5.10	21.43±2.05	0.001>	reference	
		(Stable (n=92	36.91±3.91	23.67±2.99		(0.538–1.568) 0.919	0.756
		(Increased (n=80	19.25±3.05	8.37±0.84		(1.278–3.695) 2.173	0.004
	Baseline AFP<100	(Decreased (n=15	36.09±6.43	25.40±2.53	0.003	reference	
		(Stable (n=53	35.29±3.65	28.90±4.25		(0.504–2.045) 1.015	0.967
		(Increased (n=47	24.60±4.43	12.37±3.04		(1.042–4.186) 2.089	0.038
	Baseline AFP≥100	(Decreased (n=8	16.25±2.24	13.20±2.88	0.001>	reference	
		(Stable (n=39	31.86±5.67	21.10±2.87		(0.304–1.806) 0.741	0.510
		(Increased (n=33	11.18±3.25	5.47±1.09		(1.085–6.355) 2.626	0.032
.From baseline to 6 mo	AH	(Decreased (n=19	42.25±6.41	30.43±3.67	0.009	reference	
		(Stable (n=55	32.23±3.23	23.43±2.75		(0.765–2.923) 1.495	0.239
		(Increased (n=71	27.89±3.57	17.90±3.21		(1.223–4.461) 2.336	0.010
	Baseline AFP<100	(Decreased (n=12	49.87±7.88	53.93±22.54	0.052	reference	
		(Stable (n=38	32.64±3.64	23.43±3.67		(0.850–4.984) 2.058	0.110
		(Increased (n=47	28.03±3.27	20.87±2.69		(1.155–6.564) 2.754	0.022
	Baseline AFP≥100	(Decreased (n=7	24.20±3.66	30.43±8.28	0.038	reference	
		(Stable (n=16	26.17±3.85	21.73±3.39		(0.319–2.735) 0.933	0.900
		(Increased (n=24	18.55±4.77	9.47±1.06		(0.837–5.946) 2.230	0.109
.From baseline to 12 mo	AH	(Decreased (n=16	41.12±6.80	26.73±4.43	0.854	reference	
		(Stable (n=35	34.82±3.36	27.10±3.35		(0.539–2.396) 1.137	0.736
		(Increased (n=51	38.86±4.60	27.93±2.21		(0.603–2.465) 1.219	0.581
	Baseline AFP<100	(Decreased (n=11	40.84±8.19	26.73±3.07	0.978	reference	
		(Stable (n=22	36.71±4.40	30.23±5.83		(0.397–2.458) 0.988	0.979
		(Increased (n=32	40.90±5.72	29.07±3.10		(0.452–2.472) 1.057	0.898
	Baseline AFP≥100	(Decreased (n=5	40.69±11.87	30.43±6.47	0.749	reference	
		(Stable (n=12	32.15±5.44	25.87±1.11		(0.350–5.036) 1.328	0.677
		(Increased (n=19	33.91±7.18	23.03±3.61		(0.451–5.627) 1.594	0.469
E.Change	Patients	Platelets	Overall survival ((months Mean±SE	Overall survival ((months Median±SE	Log-rank p-value	(.HR (95% C.I	HR p-value
.From baseline to 1 mo	AH	(Increased (n=30	15.55±2.57	11.60±6.14	0.016	reference	
		(Stable (n=188	26.35±2.44	13.20±2.10		(0.477–1.084) 0.719	0.115
		(Decreased (n=51	15.79±3.03	8.50±1.22		(0.698–1.800) 1.121	0.638
	Baseline AFP<100	(Increased (n=14	17.19±2.99	19.83±6.74	0.005	reference	
		(Stable (n=102	29.76±2.63	20.87±3.02		(0.331–1.084) 0.599	0.090
		(Decreased (n=25	18.32±4.90	8.50±2.05		(0.623–2.447) 1.235	0.545
	Baseline AFP≥100	(Increased (n=14	10.17±2.79	5.47±0.72	0.568	reference	
		(Stable (n=86	17.87±3.00	8.37±1.33		(0.406–1.323) 0.733	0.302
		(Decreased (n=26	13.10±2.63	7.90±1.13		(0.413–1.596) 0.812	0.545
.From baseline to 3 mo	AH	(Increased (n=13	13.69±2.90	9.87±2.88	0.002	reference	
		(Stable (n=134	35.12±3.30	21.07±1.96		(0.245–0.821) 0.448	0.009
		(Decreased (n=47	17.80±2.19	13.37±2.45		(0.397–1.441) 0.756	0.396
	Baseline AFP<100	(Increased (n=6	14.33±3.69	19.87±3.59	0.001	reference	
		(Stable (n=80	39.90±4.19	25.37±3.20		(0.146–0.811) 0.344	0.015
		(Decreased (n=27	17.56±1.99	17.17±3.43		(0.307–1.843) 0.752	0.533
	Baseline AFP≥100	(Increased (n=7	13.84±4.99	6.80±1.75	0.549	reference	
		(Stable (n=53	25.73±4.64	13.20±3.71		(0.284–1.585) 0.671	0.363
		(Decreased (n=20	17.68±4.22	10.60±1.68		(0.328–2.126) 0.835	0.706
.From baseline to 6 mo	AH	(Increased (n=11	22.77±4.70	12.97±4.33	0.629	reference	
		(Stable (n=95	34.77±3.30	23.43±1.70		(0.403–1.742) 0.838	0.635
		(Decreased (n=37	23.66±2.85	19.33±2.11		(0.466–2.285) 1.032	0.938
	Baseline AFP<100	(Increased (n=8	23.73±4.99	12.97±5.19	0.141	reference	
		(Stable (n=66	36.08±3.17	25.63±2.63		(0.283–1.564) 0.666	0.350
		(Decreased (n=22	22.08±3.25	17.90±2.35		(0.447–2.890) 1.137	0.788
	Baseline AFP≥100	(Increased (n=3	18.93±9.77	7.13±0.27	0.502	reference	
		(Stable (n=28	23.47±4.82	15.93±3.99		(0.269–5.042) 1.165	0.839
		(Decreased (n=15	22.19±3.12	27.83±10.47		(0.156–3.502) 0.739	0.703
.From baseline to 12 mo	AH	(Increased (n=12	24.70±3.05	23.20±2.60	0.074	reference	
		(Stable (n=61	46.32±4.59	30.23±4.64		(0.235–1.027) 0.492	0.059
		(Decreased (n=28	28.14±2.53	25.87±0.95		(0.363–1.754) 0.798	0.574
	Baseline AFP<100	(Increased (n=8	23.43±2.22	23.20±2.26	0.086	reference	
		(Stable (n=44	48.02±5.61	35.70±5.97		(0.168–0.946) 0.399	0.037
		(Decreased (n=12	30.65±3.82	26.73±3.54		(0.227–1.648) 0.611	0.331
	Baseline AFP≥100	(Increased (n=3	32.59±8.39	NA	0.638	reference	
		(Stable (n=17	40.36±7.65	27.83±4.85		(0.173–10.541) 1.349	0.776
		(Decreased (n=16	24.41±2.16	25.87±4.56		(0.244–15.056) 1.918	0.536

F. Change	CRP-All patients	Overall survival (months) Mean±SE	Overall survival (months) Median±SE	Log-rank p-value	HR (95% C.I.)	HR p-value
From baseline to 1 mo.	Decreased (n=8)	10.47±3.66	5.57±1.84		reference	
	Stable (n=11)	23.74±9.72	8.37±3.47	0.067	0.626 (0.234–1.672)	0.350
	Increased (n=13)	5.27±1.65	3.53±0.76		1.752 (0.712–4.316)	0.222
From baseline to 3 mo.	Decreased (n=6)	18.58±4.57	15.93±3.18		reference	
	Stable (n=3)	39.86±22.43	19.83±11.65	0.084	0.550 (0.110–2.764)	0.468
	Increased (n=12)	9.14±1.82	7.80±4.27		2.308 (0.828–6.430)	0.110
From baseline to 6 mo.	Decreased (n=4)	21.64±3.35	17.10±2.38		reference	
	Stable (n=5)	66.36±15.23	NA	0.039	0.270 (0.043–1.681)	0.161
	Increased (n=13)	18.79±4.21	11.37±2.44		1.532 (0.422–5.562)	0.516
From baseline to 12 mo.	Stable (n=5)	37.23±4.91	NA		reference	
	Increased (n=4)	19.73±1.88	17.10±3.25	0.051	6.855 (0.746–62.943)	0.086

AFP is reported in IU/mL. Abbreviations: ALBI, albumin-bilirubin grade; CRP, C-reactive protein; GGT, gamma-glutamyl transferase; HR, hazard ratio; mALBI, modified ALBI grade; PALBI, platelet-albumin-bilirubin grade; total bilirubin, total serum bilirubin.

PALBI, Platelet-ALBI score; mALBI, modified ALBI score; CRP, C-reactive protein; HR, Hazard Ratio.

**Table 4:** Associations between AFP changes and changes in other biomarkers post-TARE.

A.Baseline AFP<100 IU/mL		AFP changes at 6 months			
Changes at 6 months.		Decreased	Stable	Increased	p
Albumin	Increased	19 (41.3)a	5 (18.5)b	1 (2.9)c	0.001
	Stable	17 (37)	14 (51.9)	15 (44.1)	
	Decreased	10 (21.7)a	8 (29.6)ab	18 (52.9)b	
ALBI grade	Decreased	18 (39.1)a	4 (14.8)b	3 (8.8)b	0.003
	Stable	19 (41.3)a	18 (66.7)b	16 (47.1)ab	
	Increased	9 (19.6)a	5 (18.5)a	15 (44.1)b	
mALBI grade	Decreased	21 (45.7)a	4 (14.8)b	4 (11.8)b	0.001
	Stable	14 (30.4)a	15 (55.6)b	12 (35.3)ab	
	Increased	11 (23.9)a	8 (29.6)ab	18 (52.9)b	
PALBI grade	Decreased	17 (37)	5 (18.5)	7 (20.6)	0.273
	Stable	18 (39.1)	16 (59.3)	16 (47.1)	
	Increased	11 (23.9)	6 (22.2)	11 (32.4)	
GGT	Decreased	13 (28.3)	7 (25.9)	10 (29.4)	0.992
	Stable	21 (45.7)	12 (44.4)	14 (41.2)	
	Increased	12 (26.1)	8 (29.6)	10 (29.4)	
CRP	Decreased	5 (26.3)	2 (40)	1 (16.7)	0.719
	Stable	7 (36.8)	1 (20)	1 (16.7)	
	Increased	7 (36.8)	2 (40)	4 (66.7)	

B.Baseline AFP≥100 IU/mL		AFP change in 6th mo.			
Change in 6th month.		Decreased	Stable	Increased	p
Albumin	Increased	7 (21.2)	0 (0)	0 (0)	0.044
	Stable	17 (51.5)	1 (16.7)	6 (66.7)	
	Decreased	9 (27.3)a	5 (83.3)b	3 (33.3)ab	
ALBI grade	Decreased	4 (12.5)	0 (0)	2 (22.2)	0.183
	Stable	21 (65.6)	2 (33.3)	4 (44.4)	
	Increased	7 (21.9)	4 (66.7)	3 (33.3)	
mALBI grade	Decreased	5 (15.6)	0 (0)	2 (22.2)	0.172
	Stable	17 (53.1)	1 (16.7)	3 (33.3)	
	Increased	10 (31.3)	5 (83.3)	4 (44.4)	
PALBI grade	Decreased	7 (21.9)	1 (16.7)	2 (25)	0.232
	Stable	14 (43.8)	0 (0)	3 (37.5)	
	Increased	11 (34.4)	5 (83.3)	3 (37.5)	
GGT	Decreased	11 (34.4)	1 (16.7)	2 (22.2)	0.158
	Stable	12 (37.5)	1 (16.7)	1 (11.1)	
	Increased	9 (28.1)	4 (66.7)	6 (66.7)	
CRP	Decreased	2 (25)	0 (0)	0 (0)	0.444
	Stable	3 (37.5)	0 (0)	0 (0)	
	Increased	3 (37.5)	0 (0)	2 (100)	

Different superscript letters indicate statistically significant differences between column proportions. Abbreviations are as defined in Tables 1 and 2.

**Table 5:** Network phenotyping strategy (NPS) changes post-TARE.

A. Distribution of NPS phenotype changes.	
:(NPS Phenotype Decrease (Improvement	20.8% = 24/115
:NPS Phenotype Stable	66.1% = 76/115
:(NPS Phenotype Increase (Progression	13.0% = 15/115

B. Examples of NPS phenotype changes post-TARE and survival.			
NPS Responders (NPS decrease)		NPS Progressors (NPS increase)	
Phenotype change	Follow up (mo.)	Phenotype change	Follow up (mo.)
235	22.97	1924	4.30
225	64.27	1522	4.50
2114	44.97	1425	8.83
203	32.83	1222	3.73
198	53.60	1222	4.60
195	22.60	1222	3.50
192	33.97	1219	8.50
186	14.63	1125	3.53
185	51.93	1019	5.47
152	49.33	915	9.90
152	30.43	823	4.03
153	48.10	519	7.63
145	25.40	512	6.53
125	52.87	524	18.17
125	37.97	320	6.43
125	93.47		
125	31.17		
121	59.67		
123	11.60		
122	12.83		
118	26.63		
105	39.33		
105	37.07		
104	27.83		

C. NPS changes post-TARE and overall survival.		
	Survival (months)	
	Mean±SE	Median±SE
NPS phenotype decrease (Response)	47.76±6.39	37.07±5.22
NPS phenotype unchanged (Stable)	30.03±3.02	21.37±1.44
NPS phenotype increase (Progression)	6.65±0.98	5.47±1.25
NPS Phenotype 12 Response	60.34±14.35	NA
NPS Phenotype 12 Progression	5.08±1.16	3.73±0.55
